# Childhood trauma and violent behavior in adolescents are differentially related to cognitive-emotional deficits

**DOI:** 10.3389/fpubh.2023.1001132

**Published:** 2023-04-03

**Authors:** Stephen Katembu, Anoushiravan Zahedi, Werner Sommer

**Affiliations:** ^1^Department of Psychology, Humboldt-Universitat zu Berlin, Berlin, Germany; ^2^Neuroscience Research Center, Charité-Universitätsmedizin Berlin, Berlin, Germany; ^3^Department of Psychology, University of Muenster (Westfaelische Wilhelms-Universitaet Muenster), Münster, Germany; ^4^Department of Psychology, Zhejiang Normal University, Jin Hua, China

**Keywords:** adverse childhood experiences, cognitive control, emotional regulation, intelligence, working memory, violence

## Abstract

**Introduction:**

Converging neurobiological and epidemiological evidence indicates that exposure to traumatic events in the early stages of development, that is, adverse childhood experiences (ACEs), negatively affects the likelihood of being involved in violent behavior later in life. These problems are hypothesized to be mediated by the disruption of executive functions, in particular, the ability to inhibit inappropriate actions. Here we aimed to distinguish the contribution of inhibition in non-emotional and emotional situations (i.e., emotion regulation) and assessed the modulating influence of stress, testing Nairobi county high school students in a two-experiment study.

**Methods:**

In Experiment 1, neutral and emotional inhibition, working memory, and fluid intelligence were measured alongside questionnaires about ACE and violent behavior. Experiment 2 replicated these relations in an independent sample and assessed whether they would be aggravated after acute experimentally induced stress.

**Results:**

Experiment 1 results showed that ACE was positively related to both non-emotional and emotional inhibition; in contrast, violent behavior was only associated with deficient emotional inhibition. Experiment 2 findings showed that stress did not significantly affect the relation of ACE to non-emotional inhibition and emotion regulation; however, it increased deficits of violent participants in their ability to down-regulate emotions.

**Discussion:**

Together, results suggest that deficits in emotion regulation, especially under stressful conditions, are more critical than impairments in non-emotional inhibition in predicting violent behavior in victims of childhood trauma. These findings open perspectives toward more targeted research and interventions.

## 1. Introduction

Favorable childhood conditions offer a conducive milieu for the development of the brain that affects a wide range of psychological and cognitive components, such as inhibition, emotion regulation, attachment, self-image, and socialization (1). Successful development has been positively correlated with cognitive outcomes such as physical and mental health, happiness, and a lower likelihood of risky behavior and drug use (2). Whereas the basic foundation and general structure of the brain are provided for by genes, favorable experiences mold the neural connections that facilitate sensory, motor, and cognitive skills and behavior regulation (3), as well as emotion regulation (4). Notably, epidemiological studies have shown a high prevalence of unfavorable experiences among children (5). In the seminal ‘CDC-Kaiser Permanente Philadelphia study, Felitti et al. (6) coined the term *adverse childhood experiences* (*ACEs*) to refer to potentially traumatic experiences in the years prior to the age of 18. Over 17,000 members of a health maintenance organization in South California took part in this study, reporting their current health status, behavior, and childhood experiences. At least 52% of participants reported one or more ACE, consisting of physical, emotional, and sexual abuse, emotional or physical neglect by caregivers, or family disruption marked by household incidences such as witnessing violence (6). Notably, poor outcomes have been shown after both single or multiple exposures to ACEs (7).

Unlike adulthood trauma, childhood trauma presents more detrimental consequences due to its interaction with ongoing psycho-neurobiological development, leading to long-lasting effects. According to De Bellis (8), childhood maltreatment affects multiple densely interconnected neurobiological systems that affect EF development, as well as emotional and behavioral regulation. Adverse childhood experiences may lead to elevated levels of catecholamines and cortisol, leading to accelerated loss of neurons, delayed myelination, abnormal synaptic pruning, and slower neurogenesis. Magnetic resonance imaging (MRI) studies of maltreated children show reduced intracranial and cerebral volumes compared to controls with no history of maltreatment [for review, see (8)]. Exposure to traumatic life events activates the hypothalamus-pituitary–adrenal (HPA) axis, prompting the adrenal glands to produce glucocorticoids (9). Whereas this allostatic response (secretion of biochemicals to maintain homeostasis) is beneficial for adaptation and survival, during sustained exposure to stressful experiences in childhood, these biochemical responses lead to high allostatic load, associated with impaired brain development and functioning (10), destructive physiological and behavioral responses (11), and maladaptive affect processing and regulation (12).

Several studies have found strong associations between externalizing behavior and exposure to ACEs [e.g., (13)], with poor self-regulation as a developmental sequela of exposure to ACEs [e.g., (14)]. For example, in a longitudinal study, Widom (15) followed 1,575 children (667 in the control group) over a 20-year period after reporting childhood mistreatment. They found that experiencing abuse or neglect increased the chances of being arrested as a youth by 53%. Further, exposure to childhood abuse and neglect has been positively and significantly correlated with the likelihood of arrest for violence and adult criminality, with Reavis et al. (16) reporting nearly four times more ACEs in a group of adult male offenders than in a control group.

Whereas each adverse experience negatively impacts psycho-development, health, and behavior, individuals who record four or more ACEs have been shown to have a 4–12 fold higher chance of physical and mental health-related risks compared to those recording three or fewer ACEs (6). According to the review of Hughes et al. (17) involving 253,719 participants, multiple ACEs were shown to pose a major risk for negative health outcomes, with ACE scores equal to or greater than four representing the greatest risk. Such findings indicate a dose–response effect where every additional ACE score increases the possibility of negative physical and/or mental outcomes (18). Furthermore, Dong et al. (19) found that more than 81% of the respondents who reported a certain kind of adverse childhood experience also reported another type of childhood trauma. Clustering childhood experiences has been shown to be crucial in effective research and understanding the consequences of ACEs (20). Previous studies also suggest that there is a cumulative effect of ACEs that can be passed on through generations. Fox et al. (11) found that for every additional ACE experienced, participants’ risk of becoming a serious, violent, and chronic (SVC) juvenile offender increased. Multiple ACEs have also been shown to represent risks such as violence for the next generation (17). Additionally, Guedes and Mikton (21) found that adversity in childhood was associated with intimate partner violence among adolescents.

These results were also confirmed in several meta-analyses. For instance, Braga et al. (22) conducted a meta-analysis of studies addressing childhood adversity and juvenile antisocial tendencies and demonstrated that abuse (physical, emotional, sexual) and neglect significantly increased the chances of committing violent and antisocial acts among juveniles. A subsequent meta-analysis found an association between antisocial behavior and maltreatment endured through adolescence into adulthood (23). Whether considered cumulatively, categorically, or individually, it is apparent that ACEs are associated with violent tendencies.

As reviewed above, there is strong evidence linking childhood trauma and violent behavior. This raises the question: which psychological factors connect the two phenomena? In the present study, we consider executive functions, especially inhibition, emotion regulation, and working memory (WM), and the potential mediation of stress among adolescents.

The transition from childhood to the legal age of adulthood, that is, adolescence, is a time of rapid cognitive development (24), heightened hypothalamic–pituitary–adrenal (HPA) axis reactivity, presenting changes that are sometimes externalized as heightened stress-induced hormonal responses, correlated with high impulsivity, and deficiencies in inhibitory control (25). Some scholars have termed this period a time of storm and stress (26), and the age of all gasoline, with no brakes and no steering wheel (27). Whereas adolescence is a sensitive period for EF development, in a systematic review of 711 recent empirical studies on cognitive abilities and EFs, Baggetta and Alexander (28) found that only 8% of the papers focused on adolescents, much fewer than on children (24%), adults (32%), or older adults (20%). Even more, according to Poon (29), there is a dearth of data that compares the progressive development of hot cognition (thinking under emotional or motivational conditions) and cool cognition (thinking under minimal affective involvement) during adolescence. There is, therefore, a need for research that focuses on the interaction of ACEs, inhibition deficits, and emotional dysregulation among healthy adolescents, especially differentiating hot from cool cognition.

*Executive Functions (EFs)* represent a set of distinct and interrelated cognitive activities that aid an individual in adaptive responding and carrying out goal-oriented behavior (30). Two cognitive functions, i.e., *inhibition* and WM, are commonly considered core EFs, in which higher-order cognitive functions such as creativity, systematic decision-making, problem-solving, planning, and reasoning are anchored (30, 31). *Inhibition* is associated with the self-regulation mechanisms that hinder prepotent impulses and habitual responses inappropriate for the task at hand (31). Miyake and Friedman (32) distinguished two dimensions of inhibition: prepotent response inhibition (i.e., the ability to suppress a prepotent motor response) and interference control (i.e., the ability to resist irrelevant distractor information). In a comprehensive systematic review of studies on inhibitory control among trauma-exposed youth, van der Bij et al. (3) considered 33 studies, 12 of which measured prepotent response inhibition (e.g., go/no-go tasks), 20 measured interference control (e.g., Stroop tasks), and one study measured both. Their results showed that trauma-exposed youth had deficient inhibitory control. Notably, there was no evidence that the two dimensions of inhibition were differentially related to the experience of trauma. Daily situations such as navigating a busy street or being patient with a younger sibling requires to effectively and usually swiftly suppress an unwanted response or interfering information and focus on the task at hand by executing the appropriate action. Such situations, requiring response suppression and replacement – together termed inhibition - are mimicked or modeled in the classic colour word Stroop task. The colour word Stroop task is the most frequently used and best recognized task for testing the inhibition function. For instance, compared to healthy controls, when presented with the colour word Stroop task, patients with mild traumatic brain injury showed slowed RTs and higher error rates for incongruent conditions (33). Such evidence of the validity of the classic colour word Stroop task performance to capture inhibition has also been shown among patients with frontal lobe damage [e.g., (34)] and in violent offenders [e.g., (35)], and correlates with academic success in school [e.g., (36)].

There are conflicting findings on the correlation between WM and ACEs. WM decrements have been shown among individuals with a history of childhood maltreatment (37). However, Sheridan et al. (38) found that WM was unrelated to abuse. One possible explanation is that WM depends on the type and severity of abuse (37).

*Emotion regulation* is a concept closely related to inhibition and refers to “the extrinsic and intrinsic processes responsible for monitoring, evaluating, and modifying emotional reactions” (39). Emotion regulation is called for when emotional contexts automatically draw attention, interfering with other processes (40) and causing cognitive conflict (41). Such cognitive conflicts manifest in temporal costs in the emotional Stroop task, in which emotional stimuli interfere with response time (RT) in a manner irrelevant to the color-naming task (42). For instance, when responding to the print color of neutral (e.g., “white”) or emotional words (e.g., “death”), participants record RTs that are longer for emotional compared to neutral words (43). Recent studies have extended the Stroop task into the domain of facial expressions in which participants respond with a smile or frown to words pertaining to emotions (e.g., “HAPPY” or “ANGRY”) written over emotional faces (e.g., happy or angry), or neutral faces [e.g., (44)]. Results indicated longer RTs when word meaning and the face stimulus were incongruent (e.g., “HAPPY” word on “angry” face) compared to congruent and neutral word-face combinations. Longer RTs can be accounted for by cognitive conflicts getting in the way of behavior regulation in emotional contexts. Resolving such cognitive conflict requires deploying attentional resources, like amplifying task-relevant stimuli while ignoring irrelevant information (45).

The relationship between emotion regulation and inhibition might be nuanced. A study by Stawski et al. (46) showed that individuals with high cognitive capabilities, even when they experienced more frequent stressful daily situations, recorded lower mood changes in response to stressful situations. However, Botdorf et al. (47) found that emotional Stroop effects but not cognitive Stroop effects predicted risk-taking in a laboratory driving task. Further, low performance during “hot” (i.e., emotional) but not “cool” (i.e., cognitive) tasks has been uniquely related to emotional problems (29) and may underlie violent behavior among victims of childhood adversity. Therefore, one might suggest that people can hold impulses in check under emotionally neutral (cool) conditions, but their emotional regulation and, by extension, self-regulation systems may be compromised in affective contexts. This is especially important for understanding the relationship between ACEs and EFs since ineffective emotion or cognitive inhibition could be the underlying link between ACEs and violent behavior.

There is a dearth of research investigating how stress may aggravate existing cognitive, emotional, or behavioral deficits. When demands made on an organism exceed its regulatory capacity (real or perceived), the result is stress. When one encounters stressful life events, coping with them requires varied cognitive control processes to perceive, process, and respond to the given stressor (48). Findings on the effects of acute stress on core executive functions have been inconsistent. For instance, even though stress is generally thought to impair executive functions [e.g., (49)], it might benefit some aspects of cognition (50). According to ‘Easterbrook’s hypothesis, stress enhances selective attention by narrowing attention toward the relevant and away from distracting information, thus improving performance (51). Other findings indicate that under stress, selective attention deteriorates [e.g., (52)]. Further, Sänger et al. (53) showed that, under stress, people are more often than not distracted by interfering information. A meta-analysis by Shields et al. (50) has shown that stress impaired WM cognitive flexibility, and interference control. An explanation might be that the effects of stress on performance might be related to the baseline cognitive and emotional abilities. For instance, using the color word Stroop task, Booth and Sharma (51) found that the enhancement of selective attention under stress did not apply to participants with low WM spans as they showed less attentional control. Also, Gur and Algomb (54) found that when presented with the color word Stroop task, participants still paid attention to both relevant and irrelevant stimuli, but there were more resources allocated to boost inhibition, leading to better executive control rather than the narrowing of attention, which accounted for improved selectivity under stress. This study, therefore, also highlights the importance of baseline EFs abilities in stressful situations.

With studies showing that ACEs are associated with EFs and emotion regulation deficits [e.g., (6, 55)], the inconsistencies about stress effects on EFs may be explained by the baseline EFs abilities. For example, stress may improve inhibitory control by enhancing selective attention among individuals with well-developed EFs (51), but this may not be the case for participants with impaired EFs, such as victims of childhood adversity. Among victims of childhood maltreatment, stressful life events or socially taxing encounters may trigger violent behavior, which may lie dormant under neutral conditions.

The overarching aim of the present study is to investigate the relationship between the severity of childhood trauma and violence with deficits in inhibition, WM, fluid intelligence (Gf), and emotional regulation. After investigating the relationship between cognitive abilities and ACEs in experiment 1, we assessed the influence of acute stress on these functions in Experiment 2. Two separate samples of Kenyan adolescents from the general population with varied experiences of violent behavior and childhood trauma were used for Studies 1 and 2. Our hypotheses were (1) differential relationships exist between inhibition in non-emotional and emotional contexts with childhood trauma and violent behavior. (2) These associations will be aggravated by acute social stress.

**Figure 1:**
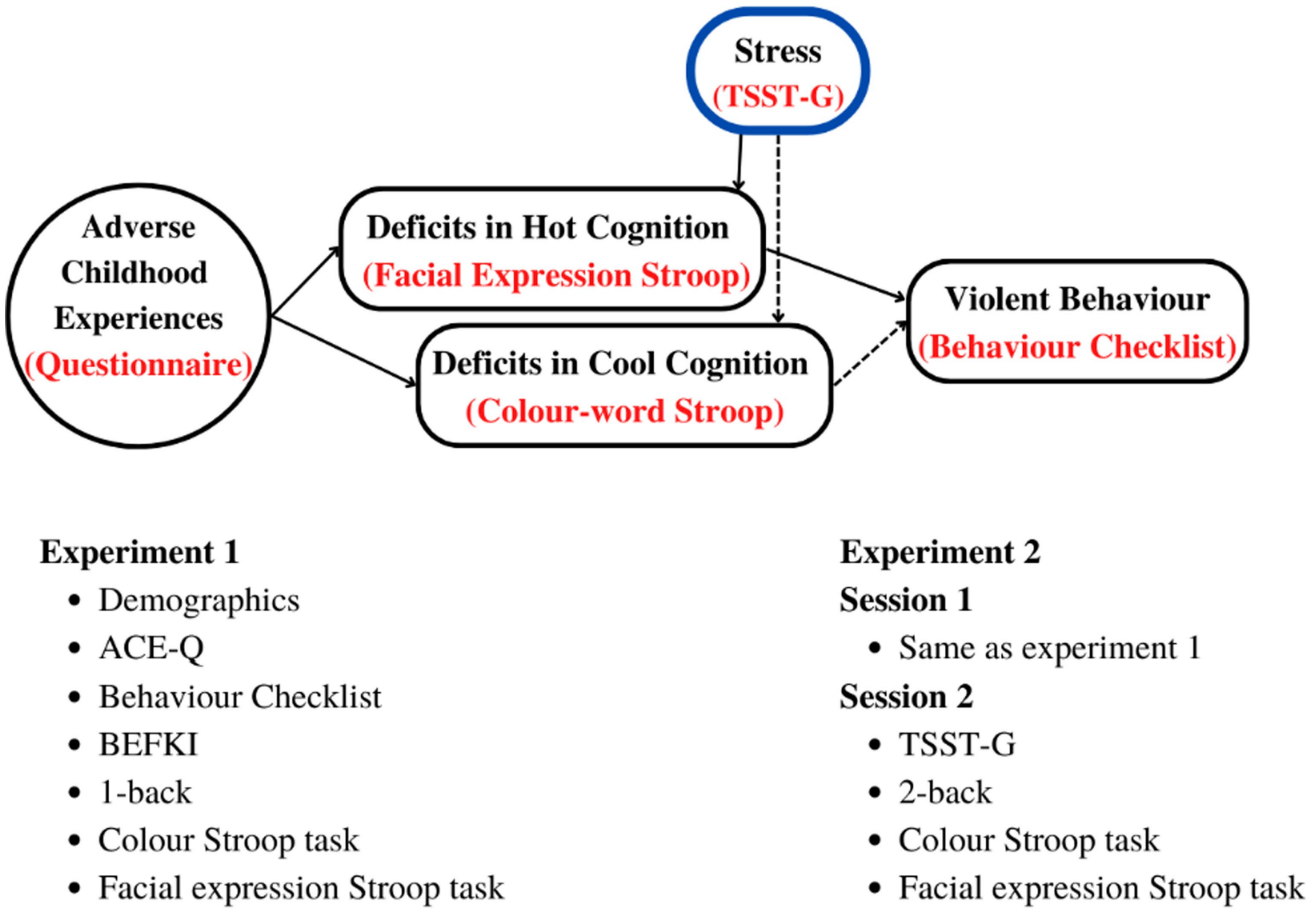
Top: Hypothetical relationship between ACEs and deficits in cool and hot EFs leading to violent behavior, and how they are affected by acute stress. Bottom: tests, questionnaires, and procedures in the order of administration. BEFKI, Berlin Test for the Detection of Fluid and Crystalline Intelligence scale; TSST-G, Trier Social Stress Task for Groups; *n*-back; Test of working memory (WM).

Figure 1 below summarises our hypothesis as well as the experiment procedures and variables as explained under methods for each of the two studies.

## 2. Experiment 1

### 2.1. Methods

#### 2.1.1. Participants

Participants were 53 male high school students in Nairobi County (*M*: 16.30 years, SD = 1.08, range = 15–18) who had covered at least ten years of schooling (*M* = 11.04, SD = 1.02); they came from medium to low-income backgrounds and attended county boarding schools. Prior to participation, written informed consent was obtained from each participant and the participant’s parent or guardian. When the data was collected, none of the participants was currently on medication, had a history of mental or neurological health disorder, or was addicted to a substance of abuse. All participants reported normal or corrected-to-normal visual acuity and normal color vision. The study was approved by Kenya’s National Commission for Science, Technology, and Innovation (NACOSTI) and the Kenyatta National Hospital-University of Nairobi – Ethics and Research Committee (KNH/UoN-ERC).

To determine the minimum sample size required to test the study hypothesis, we conducted an apriori power analysis using G*Power version 3.1.9.7 (56). To achieve 80% power for detecting a minimum effect size of 0.25 at a significance criterion of *α* = 0.05, we required a sample size (N) of 28. Thus, the obtained sample size of *N* = 53 (for the colour Stroop) and *N* = 34 (for the facial expression Stroop) was adequate to test the study hypothesis.

#### 2.1.2. General procedure

Upon arrival at the designated data collection center, participants presented a signed informed consent from a parent or guardian given to them after expressing interest in participating in the study. They then proceeded to sign an informed consent form accepting to participate in the study, followed by a short demographics questionnaire, which recorded their age, mental health history, and visual acuity.

Several tasks were administered *via* laptop computers in groups of up to 5 participants. Participants sat approximately 70 cm in front of a monitor (17 in., refresh rate: 100 Hz, resolution: 640 × 480 pixels).

#### 2.1.3. Questionnaires

The *Adverse Childhood Experience* questionnaire (ACE-Q) is a 10-item scale from the seminal ‘CDC-Kaiser Permanente study (6) and measures potentially traumatic events in the years prior to age 18. Despite possible distortions resulting from historical self-reporting-memory artifacts, ACE-Q scores remain a strong predictive measure, showing good psychometric properties with high internal consistency (Cronbach’s alpha = 0.88; 57), and a high correlation between its scores, mental and physical health (58). Participants responded with a *‘yes’* or *‘no’* to each of the ten questions, with each *‘yes’* representing a score of 1 and each *‘no’* representing a score of 0. Cumulative scores, therefore, ranged from 0 to 10.

Adolescents’ involvement in various kinds of delinquent behavior was measured using the *Self-Report Behavior Checklist* (59). The 30 items in the checklist are from five categories, i.e., noncompliance, truancy, violence, substance abuse, and stealing. In the present study, we focused on the violence category. Respondents marked in the checklist if they have a history of any of the listed items (e.g., bullying, fighting) and the frequency of such offending reported as either 1 (never), 2 (rarely), or 3 (often). This questionnaire was chosen because its validity had been ascertained and successfully applied to a Kenyan population [e.g., (59)]. Cronbach’s alpha reliability coefficient of the Self-Report Behavior Checklist was 0.827. The score per category was the average of the frequencies of engaging in corresponding behaviors. Individual involvement in violent offending was then ranked as never involved/normative behavior (1–1.45), occasionally involved (1.46–2.45), and persistently involved (2.45–3).

Fluid intelligence was assessed by means of the *Berlin Test for the Detection of Fluid and Crystalline intelligence scale (BEFKI)* for grades 8–10 (60). BEFKI is a figural reasoning scale consisting of 16 non-verbal items in the form of geometrical shapes, similar to Raven’s progressive matrices. The sequence of the shapes per item changes in line with implicit rules. Participants are required to deduce those rules and choose the next two shapes in the sequence. They are given 14 min to do that. This was used as a measure of fluid intelligence (Gf), a construct presumed to be independent of prior learning and experience (61), with the advantage that it minimizes the effects of language, with a satisfactory internal consistency (Cronbach’s alpha = 0.76). Whereas this scale assessed fluid intelligence, it has been shown to be a valid measure of general intelligence under the prescribed time restrictions. Scores are the proportion of correctly solved items.

#### 2.1.4. Experimental tasks

*The n-Back Task:* This task is a standard “executive” WM task (62) and requires participants to decide if a stimulus in a temporal sequence matches an item presented *n* steps ago (63). Our WM task presented letters in the middle of the monitor (1.6° matrix/eye). Participants responded with a left click on the mouse whenever a target was presented (matched with a preceding stimulus). For example, when doing the one-back task in a sequence B, A, A, the final “A” in that sequence would be the target because it matches the “A” that was presented immediately (one trial) before. The test consisted of 150 trials, with 20% targets and 80% distractors. A blank interstimulus interval (ISI) was set at 1,500 ms, with minimum reaction time (RT) set at 100 ms and a maximum RT at 1,200 ms. Responses that fell outside this range were categorized as missing. There was one break that allowed participants to continue by pressing the left click on the mouse. Each participant was presented with the same random letters. We used the one-back task for Experiment 1.

The *Stroop color and word test (SCWT)* (64) was programmed in Presentation software (Version 18.0, Neurobehavioral Systems, Inc., Berkeley, CA).^1^ The task used here was validated in a previous study (65). The stimuli consisted of color words (e.g., *yellow*) and non-color words (e.g., *while*) written in one of four different colors (red, green, blue, and yellow). The stimuli were either congruent (e.g., *yellow* written in yellow), incongruent (e.g., *yellow* written in red), or neutral (e.g., *while* written in yellow). The neutral words were matched in length to the color words. To avoid phonological facilitation (66), the neutral words did not have a similar first letter as any of the color words used. Stimuli were presented on a grey monitor. The response keys were associated with one of the four colors, and colored stickers were used to label them accordingly. Participants were encouraged to memorize the response keys at the beginning of the experiment. Before the presentation of each stimulus word, a fixation cross was shown for 500 ms, and a 2,000 ms duration was allowed for a response. Wrong responses or responses made after the lapse of this period (2000 ms) without a response were coded as incorrect. The interval between stimulus onset and the onset of the response key press was defined as the RT. Participants were presented with 30 to 45 practice trials at the beginning of the Stroop task. Only the practice trials provided feedback on whether a response was correct or not. There was a total of 250 experimental trials. The task was approximately 15 min, including two equally spaced breaks. As they responded to incongruent (IC), neutral (N), and congruent (C) color word combinations, the response time (RT) was the output. The difference in RT between IC and C conditions, i.e., the Stroop effect, was taken as a measure of inhibitory control, with higher Stroop effects implying lower inhibition ability. The difference in RTs between N and C represented facilitation effects, while the difference in RTs between IC and N represented inhibition effects.

**Figure 2:**
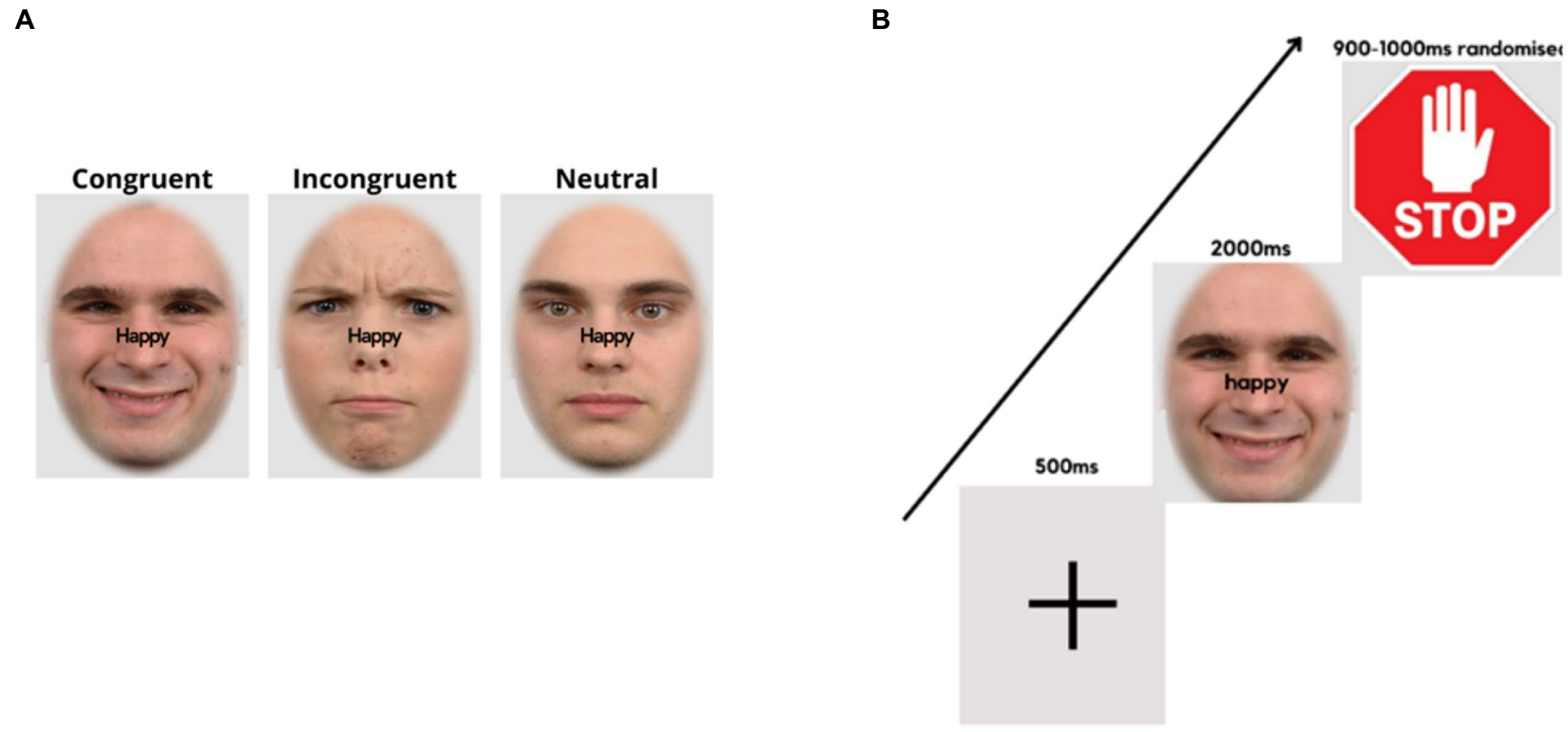
Face expression Stroop task. **(A)** Examples of stimulus presentation conditions for the face-word compound stimuli. **(B)** Trial sequence, starting with a fixation cross, followed by a word-face compound stimulus where the word demands a corresponding facial expression, and a stop signal, requiring the expression to return to neutral.

In the *emotional-expression Stroop task* (44, 67), face pictures of 60 adults (30 males) with different emotional expressions (happy, angry, neutral) were taken from the Radboud Faces Database (68). The face pictures were matched in size and brightness. The words “happy” and “angry” written in black were superimposed on the faces in the midline and at the saddle of the nose (see Figure 2A). The orthogonal combination of the words (happy, angry) with stimulus expressions (positive, negative, or neutral), yielded congruent, incongruent and neutral conditions.

The experimental procedure was controlled by “Presentation” software (version 19.0 build 11.14.16). At the top of the laptop computer monitor, a Logitech C270 video camera (video capture resolution, 720p) was mounted, which recorded the participant’s facial expressions at 25 frames per second. It was ensured that the participant’s faces were well-illuminated. Stimulus presentation/onsets were marked by a tone. Audacity software was used to locate the frame numbers of the stimulus onsets. The experiment consisted of 6 experimental conditions, each containing 80 trials, totaling 480 trials. The images were presented on a light grey background (RGB = 227/227/227), with an oval frame covering the hair, ears, and neck of the faces. Trials of all conditions appeared randomly throughout the experiment, with breaks of self-determined duration after every 120 trials. A fixation cross appeared in the center of the screen for 500 ms at the beginning of each trial, followed by a face-word compound stimulus for 2000 ms. Thereafter, a further 900–1,000 ms (randomized), a “stop” signal was presented (for 900–1,000 ms, randomized), followed by the next trial (Figure 2B).

The instructions were for participants to frown or smile as fast and accurately as possible, as per the word superimposed on the visual stimulus. Participants would then relax their facial muscles and return to a neutral expression upon presentation of the “stop” signal (see Figure 2B). Facial response so produced involved the activation of AU4 (*m. corrugator supercilii*/brow lowerer) for frowns and AU12 (*m. zygomaticus major*/lip corner puller) for smiles (69). The instructions only mentioned responses of showing a frown or a smile corresponding to the superimposed word without referring to facial muscles or action units involved. In this task, participants responded to incongruent (IC), neutral (N), and congruent (C) face-word combinations and response time (RT) was the output. The difference in response time (RT) between IC and C conditions, i.e., the Stroop effect, was taken as a measure of emotional inhibitory control, with higher Stroop effects implying lower inhibition ability. The difference in RTs between N and C represented facilitation effects, while the difference in RTs between IC and N represented emotional inhibition effects. The emotional Stroop task was validated in a previous study (67).

#### 2.1.5. Video data processing

The OpenFace software (version 2.2.0, 70) was used to analyze frame-by-frame, the video recordings. Outputs provided measures of activation for 17 AUs, including AU4 and AU12, the AUs taken to represent the target expressions, frowns, and smiles, respectively. The value increases up to 1 and above indicated activation of a given AU, while values around 0 indicate no activation. The OpenFace output text files were converted to .xlsx format in Microsoft Excel (v. 2016). Using timestamp information in the output, the data stream from audacity containing stimulus events (frame numbers) was merged with the excel files from OpenFace output. Using a procedure similar to that reported by Recio and Sommer (71), MATLAB (R2016a, The Math Works, 2016) was used to process the datasets containing information on stimulus events.

A fixed set of 90 consecutive frames was defined For each trial. This included five pre-stimulus frames and 85 post-stimulus frames, covering a time interval of 3.6 s. In each trial, a baseline correction was applied by subtracting the average intensity of AU scores over the five pre-stimulus frames (200 ms).

Data for “smile” and “frown” responses were parameterized from AU12 and AU4, respectively, to determine participants’ facial expressions and assess the correctness of responses. Target versus distractor channels were defined depending on the required response per stimulus condition. For instance, in trials where the word prompted a frown, AU4 was the target channel, and AU12 was the distractor channel. The onset, offset, and duration of target and distractor AUs were measured for each trial. The onset and offset of a given expression were measured by defining a threshold value for AU activation in each target channel for each participant. Activity onsets occurring within the first 120 ms (three frames) after stimulus onset were excluded from further analyses as they were considered fast guesses (71). When activity in the target AU channel preceded any activity in the distractor AU channel and lasted for at least seven consecutive frames above the threshold, such a response was considered as correct. All other trials were considered errors. RTs were only analyzed for correct trials (hits).

#### 2.1.6. Statistical methods

Both Correlational and categorical analyses were used. If any variable involved in a correlation was not normally distributed, Spearman’s rank correlations were used; otherwise, Pearson correlations were applied.

*Stroop effects* in the Color Stroop and the facial expression Stroop tasks were quantified as the RT difference between the incongruent and congruent conditions.

Categorical analyses were used where correlations with ACE or violence were significant. A mixed-measure ANOVA was carried out by dichotomizing these variables. With previous research showing that ACE scores equal to or greater than four (≥ 4) pose an increased risk of poor health outcomes [e.g., (17)], ACE was categorized as low if a participant *scored* ≤ 3 points and as high if ACE scores were ≥ 4, while violent behavior was categorized as normative, occasional, or frequent as described under the *self-reported behavior checklist*.

### 2.2. Results

#### 2.2.1. Descriptive statistics

Participants reported ACE scores ranging from 0 to 9 (*M* = 2.90, SD = 1.86). The most common forms of childhood trauma among the participants were physical and emotional abuse, while sexual abuse was the least prevalent (Table 1). As to be expected, ACE scores were not normally distributed (Skewness = 1.51, Kurtosis = 1.41, Shapiro–Wilk’s test for normality of distribution: *p* = 0.01).

**Table 1:** Number and frequency of ACE scores in the sample of Experiment 1 (*N* = 53).

ACE Category		Number	Frequency (%)
Abuse	Emotional	23	43.4
	Physical	27	50.9
	Sexual	2	3.8
Neglect	Emotional	17	32.1
	Physical	14	26.4
Family Dysfunction	Parental separation/divorce	14	26.4
	Domestic violence	17	32.1
	Household substance abuse	21	39.6
	Household mental illness	11	20.8
	Incarcerated family member	7	13.2

In the *Self-Report Behavior Checklist,* 50% of all participants reported normative behavior, while the others reported histories of occasional violent behavior. Accordingly, violent behavior scores were not normally distributed (Skewness = 0.72, Kurtosis = −2.80; and Shapiro–Wilk’s test normality of distribution: *p* = 0.00).

#### 2.2.2. Correlational and categorical analyses

Table 2 presents a correlation matrix across the dependent variables in Experiment 1. From ACE scores, *N* = 34 participants were defined as having low ACE, and *N* = 19 as high ACE. According to the categorization rules for the *self-reported behavior checklist*, *N* = 27 participants were defined as reporting normative behavior, and *N* = 26 as reporting occasional violence. We did not have any participants who were in the frequent violence category. These participant categories were used as group factors in ANOVAs of the dependent variables.

**Table 2:** Correlation matrix for all dependent variables in Experiment 1.

	ACE	Violence	Gf	Working memory	Color Stroop	Facial Stroop SMILES	Facial Stroop FROWNS
Violence	0.380**						
Gf	−0.126	−0.184					
Working Memory	−0.012	−0.230	0.094				
Color Stroop	0.356**	0.237	−0.088	−0.058			
Facial Stroop, SMILES	0.342*	0.617**	−0.082	−0.329	0.242		
Facial Stroop, FROWNS	0.319	−0.052	0.237	−0.055	0.239	0.338	
Facial Stroop, COMBINED	0.448**	0.320	0.119	−0.116	0.291	0.701**	0.864**

**p < 0.01 (2-tailed). *p < 0.05 (2-tailed).

#### 2.2.3. Color Stroop task

*The Stroop effects* were found to be positively correlated with the cumulative ACE scores [*r*(51) = 0.36, *p* = 0.01], while the correlation of the color Stroop effect with violence scores was also positive but only a trend [*r*(51) = 0.24, *p* = 0.09]. There were no significant correlations with the facilitation component (C-N) [*r*(51) = 0.26, *p* = 0.18] or inhibition components (IC-N) of the Stroop effect [*r*(51) = 0.25, *p* = 0.21].

**Figure 3:**
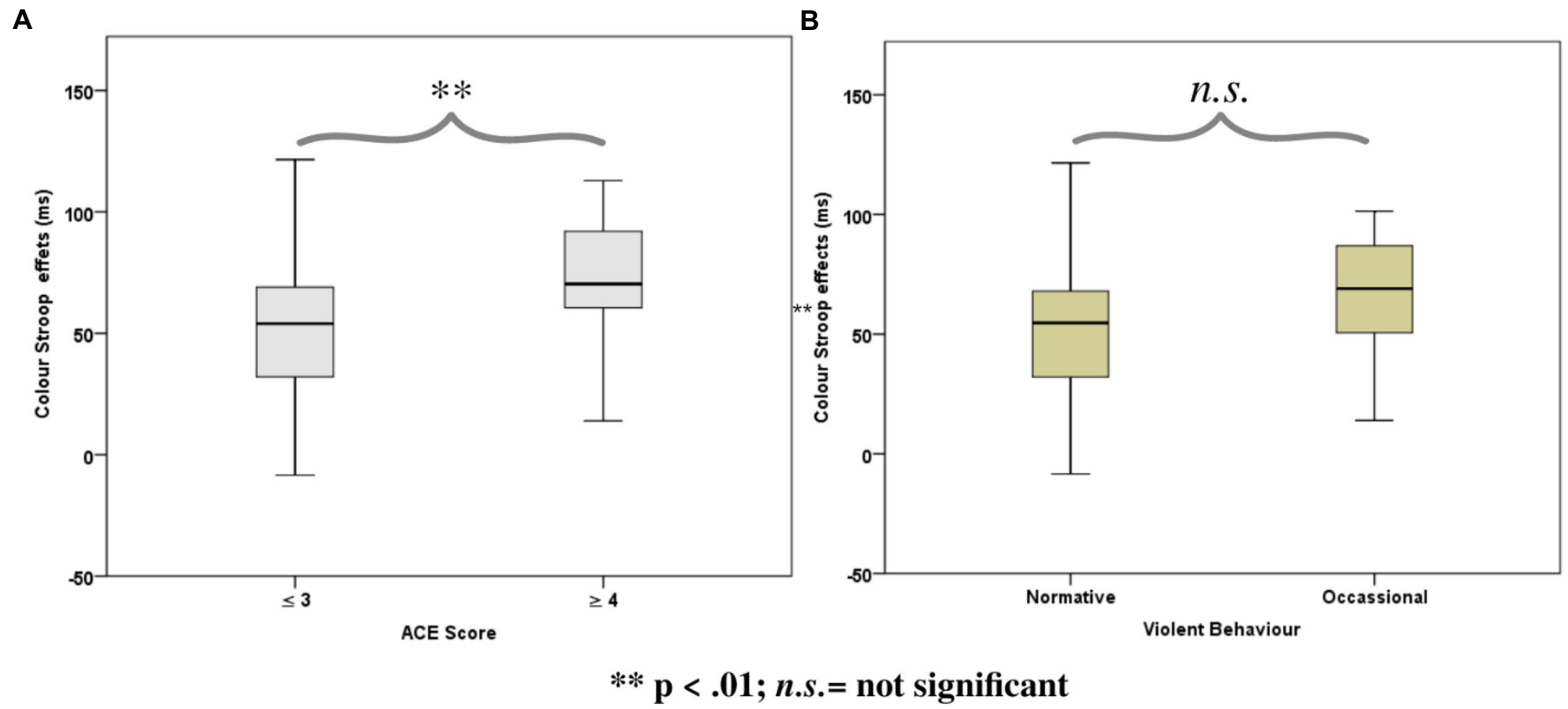
Boxplots for the color word Stroop effects (incongruent minus congruent), separated for the Group factors according to **(A)** Adverse Childhood Experience (ACE) and **(B)** Self-reported Violent Behavior.

ANOVAs of *RTs* in the Color Stroop task, with a group factor *ACE* or *Violence* with repeated measures of congruency (C, N, IC), and conditions are presented in Table 3. Both ANOVAs showed strong effects of factor congruency. The *ACE* factor interacted with *congruency*. As shown in Figure 3A, relative to ACE scores ≤3, an ACE score ≥ 4 significantly increased a participant’s Stroop effects. The interaction of ACE and the color Stroop effect appeared to be mainly due to the inhibition component of the Stroop effect. The group factor *violence* yielded no interaction effect with *congruency* (Figure 3B).

**Table 3:** Experiment 1: ANOVA results for group factors ACE and Violence with repeated measures on Congruency (congruent, incongruent, neutral) for the color word Stroop and the facial expression Stroop tasks.

Group factor	Source	df	*F*	*p*	*η* ^2^
Color word Stroop					
ACE	Congruency (C)§	2, 102	155.54**	0.00	0.75
	C × ACE	2,102	5.97**	0.00	0.11
	#Facilitation: C	1,51	79.71**	0.00	0.61
	#Facilitation: C × ACE	1,51	1.48	0.23	0.03
	#Inhibition: C	1,51	131.29**	0.00	0.72
	#Inhibition: C × ACE	1,51	7.59*	0.03	0.13
Violence	C	2,102	139.17**	0.00	0.73
	C × Violence (V)	2,102	1.31	0.27	0.03
Facial expression Stroop					
ACE	C	2,64	34.28**	0.00	0.52
	C × ACE	2,64	2.17	0.12	0.06
	Response (R)	1,32	16.05**	0.00	0.33
	R × ACE	1,32	0.16	0.70	0.01
	C × R	2,64	3.11*	0.05	0.37
	C × R × ACE	2,64	0.05	0.95	0.00
	#SMILE: C	2,64	17.86**	0.00	0.36
	#SMILE: C × ACE	2,64	1.18	0.31	0.04
	#FROWN: C	2,64	21.49**	0.00	0.40
	#FROWN: C × ACE	2,64	1.08	0.35	0.03
Violence	C	2,64	35.20**	0.00	0.52
	C × V	2,64	3.08*	0.05	0.09
	R	1,32	16.18**	0.00	0.34
	R × V	1,32	0.41	0.52	0.01
	C × R	2,64	3.33*	0.04	0.09
	C × R × V	2,64	2.36	0.10	0.07
	#SMILES: C	2,64	19.28**	0.00	0.38
	#SMILES: C × V	2,64	3.81^†^	0.06	0.11
	#FROWNS: C	2,64	21.47**	0.00	0.40
	#FROWNS: C × V	2,64	1.06	0.35	0.03

*p < 0.05; **p < 0.01; ^†^*p* < 0.1. The significant effects are shaded grey. #Post-hoc test; §The F-values for the same within-subject test, e.g., Congruency, may slightly vary when the participants are dichotomized according to ACE and violence scores into groups of different sizes because the error variance then slightly differs.

#### 2.2.4. Facial expression Stroop task

*Stroop effects* for the facial expression Stroop task (averaged across smiles and frowns) did not correlate significantly with cumulative *ACE* or *violence* scores (see Table 2). However, *Stroop effects* for smile responses were significantly correlated with cumulative *ACE* [*r*(32) = 0.34, *p = 0*.048] and *violence* scores [*r*(32)= 0.68*, p* = 0.00], whereas frown-response *Stroop effects,* when correlated with ACE, yielded only a trend [*r*(32) = 0.32, *p* = 0.06].

**Figure 4:**
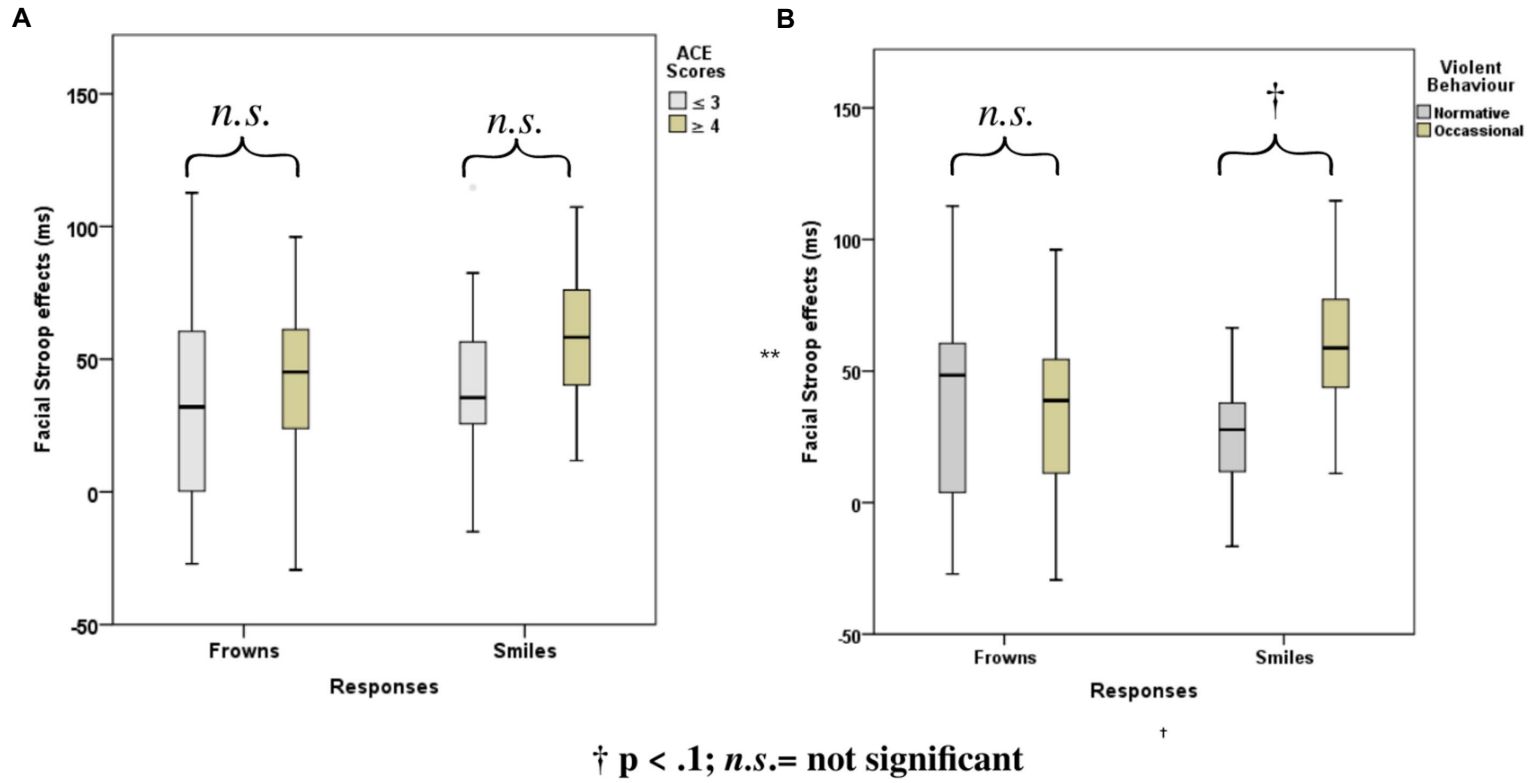
Mean Face Stroop effects for frown and smiled responses for the group factors **(A)** *ACE* and **(B)** *violence*.

ANOVAs with group factors *ACE* or *Violence* and repeated measures on congruency (C, N, IC) and response (happy, angry) yielded the main effects of *congruency, response,* and the interaction of *congruency* and *response* (Table 3). As shown in Figure 4, participants with low ACE scores (≤ 3) showed significantly smaller Stroop effects (IC-C) than participants with high ACE scores (≥ 4) for both smiles and frowns.

The ANOVA with group factor *violence* and repeated measures on congruency (C, N, IC) and response (happy, angry) yielded an interaction of *congruency and violence* (Table 3). As shown in Figure 4, participants who reported occasional violence recorded significantly higher Stroop effects compared to those with normative scores for violent behavior.

ANOVAs of *accuracy rates* showed no significant experimental effects beyond what we had found with the RTs (See Supplementary Table A1).

#### 2.2.5. Working memory and fluid intelligence

Fluid Intelligence and WM were considered control variables for which we did not expect any relationship between ACE and non-normative behavior. Indeed, *n*o significant correlations were found between cumulative ACE scores and WM [*r*(51) = −0.01, *p* = 0.93] or between violence and WM [*r*(51) = −0.23, *p* = 0.10]. Further, no significant correlations were found for cumulative ACE scores and Gf [*r*(51) = −0.13, *p* = 0.37], or for violence and Gf [*r*(51) = −0.18, *p* = 0.19].

### 2.3. Discussion

The aim of Experiment 1 was to examine how ACEs and violent behavior relate to various dimensions of EFs. To reach this aim, cognitive and emotional inhibition, fluid intelligence, and WM were measured in a sample of 53 male high school students in Nairobi with various experiences of ACEs. The results showed a significant relationship between ACEs and aggression with cognitive and emotional inhibition but not with fluid intelligence and WM.

Together the results of Experiment 1 confirm that ACEs are associated with cognitive inhibition deficits. Here we also show that deficiencies in emotion regulation are related to ACEs and represent a risk factor for violent offending.

## 3. Experiment 2

The aim of Experiment 2 was, first, to replicate the findings of Experiment 1, especially with respect to the novel facial expression Stroop task with the same procedure. Notably, as we did not find any significant correlation between WM and ACEs or violent behavior, we decided to replace the one-back task with a more demanding two-back version. This change would address whether the previous null results were related to ceiling effects (72) or to the specificity of ACEs’ impact on cognitive and emotional inhibition.

The second and more critical question of Experiment 2 was whether stress alters the relationships observed in Experiment 1. Here, we expected that stress would further aggravate any deficits in inhibition, especially concerning emotional responses. To address these two aims, Experiment 2 was conducted in two sessions, the first of which was identical to Experiment 1. The second session, however, started with a stress-inducing social task, followed by the same cognitive tasks used in the first session.

### 3.1. Methods

#### 3.1.1. Participants

From a larger group of 150 high school male students who filled the ACE-Q, we selected 62 participants (mean age: 16.45 years, SD = 1.03, range 15–18) who had covered at least ten years of schooling (*M* = 10.44, SD = 1.22), attempting to oversample participants with ACE scores ≥4. As in Experiment 1, participants came from medium to low-income backgrounds and attended county boarding schools. Written informed consent was obtained from each participant and their parent or guardian. When the data was collected, none of the participants was ill, on medication, had a history of mental or neurological health disorder, or was addicted to a substance of abuse. All participants reported normal or corrected-to-normal visual acuity and passed a color vision test.

To increase the power (from 80 to 95%) for detecting a minimum effect size of 0.25 at a significance criterion of *α* = 0.05, we conducted an apriori power analysis using G*Power version 3.1.9.7 (56). A sample size (N) of 44 would be required. Thus, the obtained sample size of *N* = 62 was *adequate* to confirm the study hypothesis.

#### 3.1.2. Stress induction protocol

In order to induce stress, we used the Trier Social Stress Task (TSST), which is considered a gold standard for inducing acute psychosocial stress in humans (73); here, we used the group format of this task, the *TSST-G* (74). The TSST-G engages participants in public speaking and public mental arithmetic, with explicit elements of social evaluation and uncontrollability. Such tasks have been shown to arouse the hypothalamic–pituitary–adrenal axis – a core stress response system – and the autonomic nervous system among healthy humans in laboratory settings (75). The stress manipulation with the TSST-G (74) consists of three phases. In *Stage I,* a ten-minute preparation and anticipation period, participants receive instructions pertaining to their tasks and are given pencils and paper to prepare a speech to convince a selection committee about their suitability for a job of their choice. The instructions end with informing the participants about an unspecified task to come later. *Stage II* consists of a 12-min mock job interview as a public speaking task, where participants are requested to introduce themselves for two minutes to a selection committee of two seated in front of the group. The two evaluators are presented to the participants as experts in non-verbal behavior and are to withhold verbal or non-verbal feedback. However, they prompt the participants to continue talking and ask some prepared questions every time the participants fall silent. *Stage III* consists in an eight-minute arithmetic task (the previously announced “unspecified task”). Here participants are required to serially subtract a number (e.g., 16) from a larger starting point (e.g., 4,858). Different numbers are given to each participant. In the event that a participant makes a mistake, they are stopped by a committee member and required to start again from the beginning. Each participant is given up to 80 s of this task. In Stages II and III, participants perform the tasks one by one in turn in the presence of all other participants. Stress effects due to the TSST-G procedure have been shown by von Dawans et al. (74), who demonstrated increases in cortisol levels, heart rate, and subjective stress experience. More generally, the effectiveness of uncontrollability and social-evaluative threat (such as in the TSST-G used here) in inducing a two- to three-fold release of cortisol in 70–80% of participants has been documented in a meta-analysis of 208 laboratory studies by Dickerson and Kemeny (76).

All tasks and questionnaires used in Experiment 2 were identical to Experiment 1, except for the 2-back task replacing the 1-back task, as explained above. Immediately after stress induction, participants repeated the three experimental tasks: (1) the two-back (WM) task, (2) the color Stroop, and (3) the facial expression task. At the end of the experiment, participants were briefed about the experiment’s aims and goals and that all procedures were for experimental purposes.

#### 3.1.3. Statistical methods

As described in Experiment 1.

### 3.2. Results

#### 3.2.1. Self-reported ACEs

The ACE scores ranged from 0 to 10 (*M* = 3.34, *SD* = 2.51); 51.6% of respondents had experienced four or more ACEs. Table 4 shows the frequencies of the ACE scores across the three categories. As to be expected, ACE scores were not normally distributed (Skewness = 0.41, Kurtosis = −1.36, Shapiro–Wilk’s test for normality of distribution: *p* = 0.01).

**Table 4:** Frequency of ACEs in the surveyed population.

ACE category		Number	Frequency (%)
Abuse	Emotional	32	51.6
	Physical	30	48.4
	Sexual	12	19.4
Neglect	Emotional	23	37.1
	Physical	6	9.7
Family Dysfunction	Parental separation/divorce	18	29.0
	Domestic violence	15	24.2
	Household substance abuse	22	35.5
	Household mental illness	11	17.7
	Incarcerated family member	13	21.0

#### 3.2.2. Violent behavior

Slightly more than half of the participants (56.5%) reported normative behavior (i.e., had not been involved in violent behavior), while 43.5% reported occasional involvement in violent behavior. As to be expected, violent behavior scores were not normally distributed (Skewness = 0.72, Kurtosis = −2.80, Shapiro–Wilk’s test for normality of distribution: *p* = 0.00).

#### 3.2.3. Correlational and categorical analyses

Table 5 presents the correlation matrix for all dependent variables in Experiment 2. If any variable involved in a correlation was not normally distributed, we used Spearman’s rank correlations; otherwise, Pearson correlations were applied. Further, where correlations were significant, a split ANOVA was carried out for the two independent variables categorized in the same way as in Experiment 1: *ACE* ≤ 3 (low) versus ≥4 (high) and *violence, normative* versus occasional.

**Table 5:** Correlation matrix for all dependent variables in Experiment 2.

Correlations												
	ACE	Violence	Gf	WM Pre-stress	WM Post-	Color Stroop Pre-stress	Color Stroop Post-stress	Facial Stroop Smiles Pre-stress	Facial Stroop Smiles Post-stress	Facial Stroop Frowns Pre-stress	Facial Stroop Frowns Post- stress	Facial Stroop Pre-stress
Violence	0.358^**^											
Gf	−0.181	−0.009										
WM Pre-stress	−0.007	−0.020	0.033									
WM Post-stress	−0.073	−0.117	−0.024	0.346^**^								
Color Stroop Pre-stress	0.251^*^	0.234	0.118	0.396^**^	0.204							
Color Stroop Post-stress	0.284^*^	0.120	0.131	−0.009	0.187	0.280^*^						
Facial Stroop Smiles Pre-stress	0.259^*^	0.219	0.179	0.175	0.140	0.388^**^	0.308^*^					
Facial Stroop Smiles Post-stress	0.411^**^	0.289^*^	−0.032	0.115	0.054	0.163	0.388^**^	0.385^**^				
Facial Stroop Frowns Pre-stress	0.168	−0.064	0.017	−0.083	−0.069	−0.104	−0.200	0.141	0.095			
Facial Stroop Frowns Post- stress	0.168	0.118	−0.078	0.031	−0.175	0.101	0.078	0.082	0.229	0.204		
Facial Stroop Pre-stress	0.288^*^	0.139	0.134	0.073	0.063	0.247	0.055	0.726^**^	0.296^*^	0.735^**^	0.219	
Facial Stroop Post-stress	0.362^**^	0.232	−0.060	0.099	−0.091	0.156	0.249	0.268^*^	0.748^**^	0.207	0.794^**^	0.330^**^

**p < 0.01(2-tailed). *p < 0.05 (2-tailed).

#### 3.2.4. Color Stroop task

When the effects (incongruent minus congruent) from the color Stroop task were subjected to a Spearman’s rank correlation, correlations were modestly positive with cumulative *ACE* scores, both pre-stress (*r* = 0.25, *p* = 0.05) and post-stress (*r* = 0.28, *p* = 0.03). However, correlations were not significant with violence (See Table 5).

The RTs in the color Stroop task were subjected to ANOVAs with split-group factors *ACE* and *Violence*, with repeated measures on congruency (C, N, IC) and Stress (pre-stress and post-stress). Both ANOVAs showed strong main effects of congruency (Table 6).

**Table 6:** Experiment 2: RTs split ANOVA for congruent, incongruent, and neutral conditions for the SCWT and the facial expression Stroop tasks before and after stress induction.

Group factor	Source	df	*F*	*p*	*η* ^2^
Color word Stroop					
ACE	Congruency (C)	2,120	206.58**	00	0.78
	C × ACE	2,120	4.71*	0.01	0.07
	Stress (S)	1,60	0.02	0.89	0.00
	S × ACE	1,60	2.57	0.11	0.04
	C × S	2,120	2.26	0.11	0.04
	C × S × ACE	2,120	0.38	0.69	0.01
	#Facilitation: C	1,60	46.91**	0.00	0.44
	#Facilitation: C × ACE	1,60	2.00	0.16	0.03
	#Inhibition: C	1,60	158.11**	0.00	0.87
	#Inhibition: C × ACE	1,60	2.64	0.11	0.04
Violence	C	2,120	201.43**	0.00	0.77
	C × Violence (V)	2,120	2.46^†^	0.09	0.04
	S	1,60	0.03	0.87	0.00
	S × V	1,60	0.46	0.50	0.01
	C × S	2,120	2.34	0.10	0.04
	C × S × V	2,120	1.43	0.24	0.02
Facial expression Stroop					
ACE	C	2,120	217.74**	0.00	0.78
	C × ACE	2,120	8.87**	0.00	0.13
	S	1,60	0.11	0.92	0.00
	S × ACE	1,60	1.65	0.20	0.03
	R	1,60	0.40	0.53	0.01
	R × ACE	1,60	0.17	0.69	0.00
	C × S	2,120	4.45*	0.01	0.07
	C × S × ACE	2,120	0.80	0.45	0.01
	C × R	2,120	2.37	0.10	0.04
	C × R × ACE	2,120	0.43	0.65	0.01
	R × S	1,60	4.99*	0.03	0.08
	S × R × ACE	1,60	0.05	0.82	0.00
	C × S × R	2,120	2.51^†^	0.09	0.04
	C × S × R × ACE	2,120	0.71	0.50	0.01
	#Facilitation: C	1,60	64.86**	0.00	0.52
	#Facilitation: C × ACE	1,60	4.56	0.12	0.07
	#Inhibition: C	1,60	132.69**	0.00	0.69
	#Facilitation: C × ACE	1,60	3.50	0.21	0.69
Violence	C	2,120	199.84**	0.00	0.77
	C × V	2,120	1.88	0.16	0.03
	R	1,60	0.41	0.53	0.34
	R × V	1,60	0.00	0.99	0.00
	S	1,60	0.11	0.74	0.00
	S × V	2,64	2.19	0.15	0.04
	C × R	2,120	3.00*	0.05	0.05
	C × R × V	2,120	3.80*	0.03	0.06
	C × S	2,120	5.62*	0.01	0.09
	C × S × V	2,120	3.37*	0.04	0.05
	R × S	1,60	4.52*	0.04	0.07
	R × S × V	1,60	0.71	0.40	0.01
	C × S × R	2,120	2.01	0.14	0.03
	C × S × R × V	2,120	1.522	0.22	0.03
	#SMILES: C	2,120	113.34**	0.00	0.65
	#SMILES: C × V	2,120	6.04**	0.00	0.10
	#SMILES: C × S	2,120	9.24**	0.00	0.13
	#FROWNS: C	2,120	86.30**	0.00	0.59
	#Facilitation: C	1,60	60.89	0.00	0.50
	#Facilitation: C × V	1,60	0.18	0.81	0.00
	#Facilitation: C × R × V	1,60	1.27	0.78	0.02
	#Inhibition: C	1,60	133.66	0.00	0.69
	#Inhibition: C × R × V	1,60	6.58*	0.04	0.10
	#Facilitation: C × V	1,60	2.19	0.42	0.04

*p < 0.05; **p < 0.01; ^†^*p* < 0.1. The significant effects are shaded grey. #Post-hoc tests (only significant results are reported).

**Figure 5:**
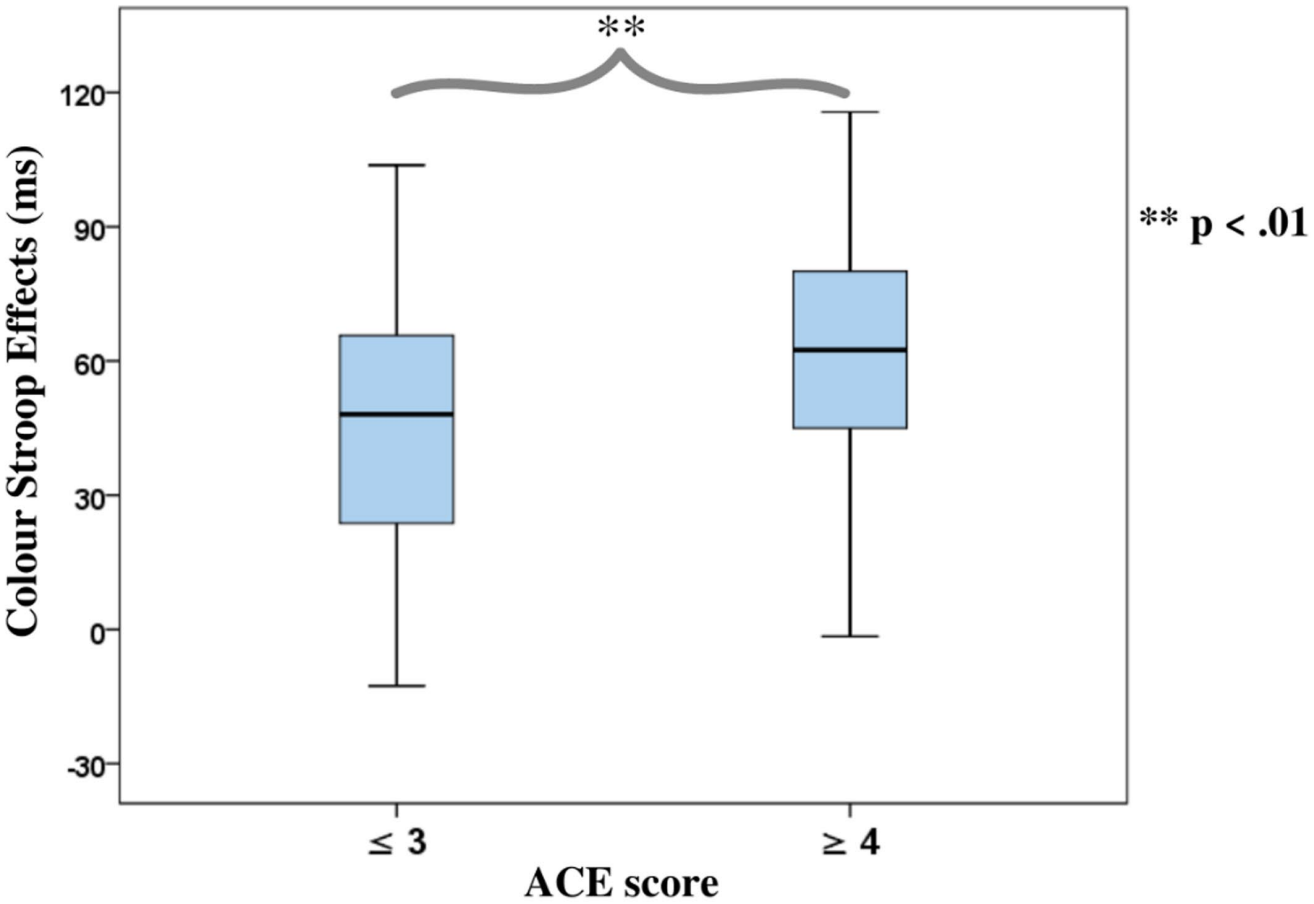
Mean colour word Stroop effects, separated for the ACE-related participant categories.

For the group factor *ACE,* ANOVA replicated the two-way interaction with *congruency* and *ACE*. Interactions with stress failed significance. As shown in Figure 5, the Stroop effect was significantly larger in the high ACE than in the low ACE group. For the group factor *Violence,* ANOVA (Table 6) found a weak trend for the two-way interaction effect of *congruency* and *violence*.

Accuracy rates were largely in line with the RTs results (see Supplementary Table A2).

#### 3.2.5. Facial expression Stroop task

Stroop effects for smile responses (incongruent minus congruent) yielded a positive and modestly significant Spearman’s rank correlation with cumulative ACE scores, pre-stress (*r* = 0.26, *p* = 0.04), and medium-sized correlations post-stress (*r* = 0.46, *p* = 0.00). Smile response *Stroop effects* pre-stress correlated weakly with measures of *violence* (*r* = 0.22, *p* = 0.09), but were modest in size and significantly correlated post-stress (*r* = 0.29, *p* = 0.02). Frown responses yielded no positive correlations (Table 5).

Separate ANOVAs of RTs with group factors ACE and Violence, including repeated measures on congruency (C, N, IC), response (happy, angry), and stress (pre-stress and post-stress) (see Table 6), both yielded the main effects of congruency and Response, and interactions of Response*Stress and Congruency*Stress, confirming the effectiveness of the stress procedure.

The ANOVA for group factor *ACE* also indicated a two-way interaction with Congruency and a weak trend for the three-way interaction Congruency*Stress*Response (see Table 6).

**Figure 6:**
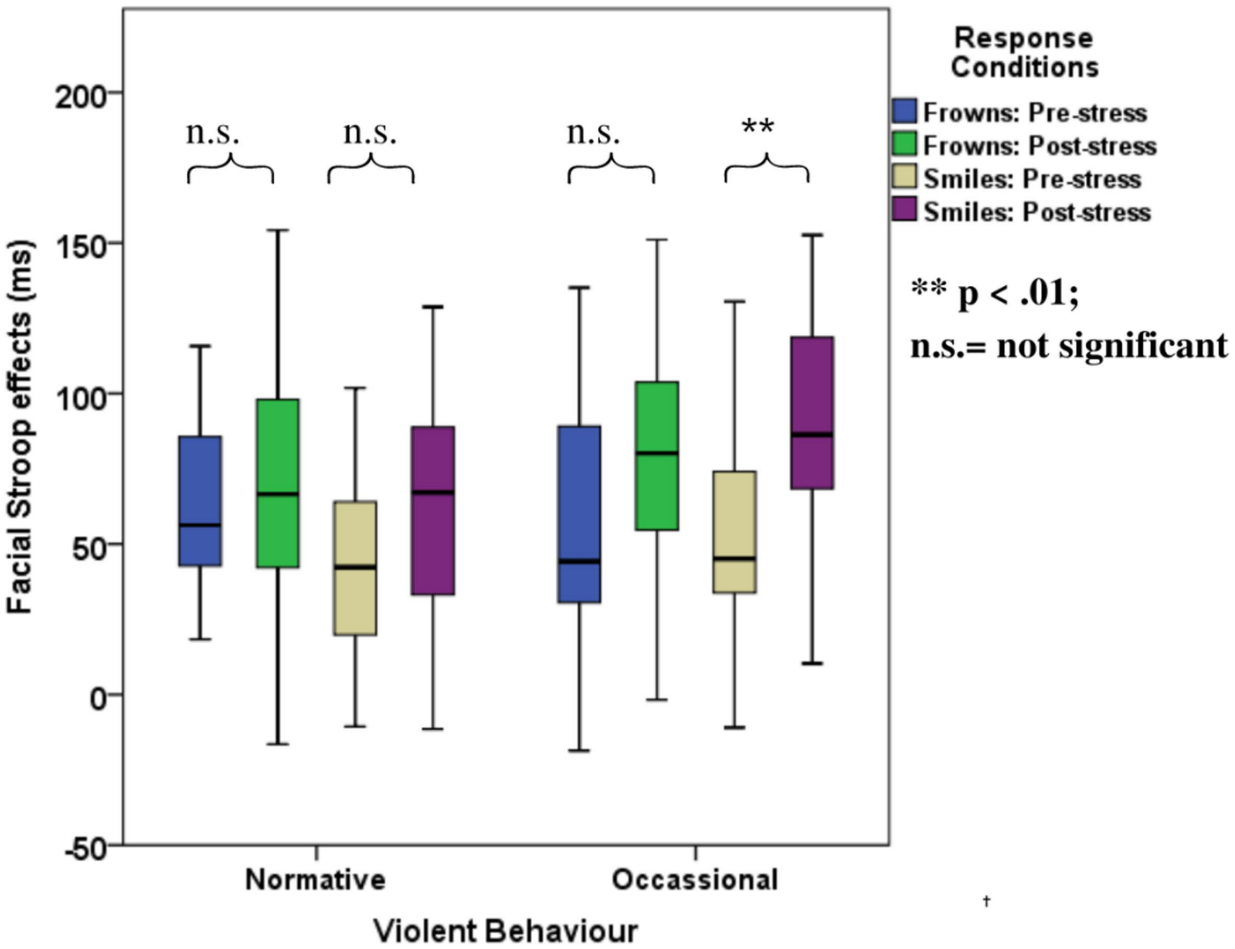
Boxplots for the facial expression Stroop task for the violence-related group, pre- and post-stress conditions.

The ANOVA with violence as a group factor yielded additional three-way interactions of congruency*response*violence and congruency*stress*violence. Interaction effect seemed to be associated more with smile responses as posthoc analysis of the first interaction yielded significant interaction effects of congruency*violence and congruency*stress for smile response but not frown responses (see Table 6). Participants who reported occasional violence recorded significantly higher Stroop effects compared to those with normative scores. Such differences in Stroop effects were more pronounced for the smile responses compared to the frown responses. As indicated by the second interaction, which is illustrated in Figure 6, the congruency effect (for both smiles and frowns) was more pronounced after stress than before stress.

#### 3.2.6. Working memory and fluid intelligence

As in Experiment 1, no significant correlations were found between cumulative ACE scores and Gf (*r* = −0.18, *p* = 0.16) or for violence and Gf (*r* = −0.01, *p* = 0.95). Similarly, cumulative ACE scores did not significantly correlate with WM measured pre-stress (*r* = −0.01, *p* = 0.96) or post-stress (*r* = −0.07, *p* = 0.57), nor was violence significantly correlated with WM pre-stress (*r* = −0.02, *p* = 0.88) and post-stress (*r* = −0.11, *p* = 0.36).

### 3.3. Discussion

The first aim of Experiment 2 was to replicate the findings of Experiment 1 regarding the relationship of inhibition in non-emotional and emotional contexts to childhood trauma and violent behavior. As will be discussed below, the replication attempt was successful.

Second, and more importantly, we sought to establish whether these relationships are aggravated by acute social stress. Independent of participant grouping, stress increased both Stroop effects in general. This finding demonstrates that the stress induction procedure worked as expected and can be considered a manipulation check for the effectiveness of the TSST-G. However, stress did not affect the relationships between ACEs or violent behavior with any measured non-emotional function. The color Stroop effects were not modulated by stress, nor was the larger congruency effect for high ACE participants increased by stress.

However, emotional Stroop effects were affected by stress. In Experiment 1, we found that responses to smile trials are better predictors of violent behavior compared to frown trials. Interestingly, in Experiment 2, the stress manipulation confirmed the special status of emotion regulation for smile responses in violent behavior. That is, the correlation of the overall facial Stroop effect with violence was significant only in the post-stress condition. In the categorical analysis, the interaction of Congruency and Violence was significantly modulated by stress, specifically due to an increase in the facial Stroop effect for smiles but not frowns.

Together, the results of Experiment 2 replicated Experiment 1 in a separate sample. Second, our findings showed that stress explicitly affects the relationship between emotion regulation and violent behavior.

## 4. General discussion

The present study addressed two overarching questions, (1) the relationship of different forms of cognitive processes to childhood trauma and violent behavior and (2) the modulation of these relations by experimentally induced stress. The first question was investigated in Experiment 1 and replicated in Experiment 2, while the second question was studied in the second experiment only.

In both experiments, participants were male students of county boarding schools from modest to poor socio-economic backgrounds. In Sample 1, 64.2% of participants had experienced at least three childhood adverse events, compared to 48.4% in the positively selected second sample.

### 4.1. Ace but not violence are related to non-emotional inhibition

In both experiments, we found a significant relationship between the color Stroop effect with ACE but not violence. ACE scores equal to or greater than four have been shown to pose an increased risk of poor health outcomes [e.g., (17)]. Accordingly, we categorized participants based on these criteria into two groups with high and low ACEs. The categorical analysis in both experiments showed that individuals with four or more traumatic events in childhood showed reduced inhibitory control, that is, larger Stroop effects. According to the separate ANOVA of the interference and facilitation components, the deficit of high ACE participants might be attributed to the interference component, indicating that the ability of participants to subdue distracting information is diminished by ACEs (65). However, one should treat this conclusion cautiously, as this difference between interference and facilitation was significant only in Experiment 1 but was only a trend in Experiment 2.

Together, the color word Stroop task indicated inhibitory control decrements to the degree that an individual experienced adversity in childhood. Such findings are in line with earlier studies showing that childhood trauma might result in deficient inhibition control [e.g., (3)]. Additionally, Stroop effects, as reviewed by van der Bij et al. (3), are likely to be increased in individuals that had been exposed to trauma early in life. Even among healthy participants, emotional neglect and physical abuse have been shown to be correlated with decreased cognitive control, as well as deficits in executive functioning in adulthood (37). These findings are in line with previous studies [e.g., (77)] and strongly suggest that accumulated ACEs impair cognitive functions in non-emotional conditions. The findings from both experiments that inhibition under non-emotional conditions are not significantly correlated with violence may indicate that the colour Stroop reflects a (partially) different kind of inhibition that is less related to self-regulation and affect regulation and a case of cool rather than hot EFs [e.g., Poon (29)]. Notably, although the association between non-emotional inhibition (colour Stroop) and violence is not significant, it is in the same (positive) direction as the significant association between hot inhibition (facial expression Stroop) and violence-related group.

### 4.2. Ace and violence are both related to deficits in emotion regulation

Previous studies [e.g., (47)] suggest that in affective contexts, people might not be able to control their impulses even if they can do so in emotionally neutral conditions. Therefore, besides the cognitive Stroop task, we used the facial Stroop task that assesses response inhibition in an emotional context and can be seen as tapping into emotion regulation. In the facial Stroop task, a verbal prompt to smile or frown was presented superimposed on an emotional face (44, 67). Congruency effects in this task may be explained by facial mimicry, a motor action resulting from the spontaneous imitation of the facial expressions of others [e.g., (78)].

Although the color word Stroop and facial Stroop tasks share the property of overlapping relevant and irrelevant information, they differ in their relationship to interpersonal emotion. Whereas the color word Stroop task is purely cognitive and non-emotional, the face Stroop task requires an emotional facial expression in response to a word designating an emotional expression (smile, frown), while a background face displays a facial expression (67). The possibility that the congruency effects in these tasks tap into different aspects of control (cognitive vs. emotional) is supported by the absence of correlations between the color Stroop effects and face Stroop effects in both experiments.

In both experiments, the correlational analysis showed higher ACE scores were accompanied by larger overall facial Stroop effects, that is, diminished emotion regulation. However, in the categorical analysis, we found an interaction between Congruency and ACE only in Experiment 2 but not in Experiment 1. Based on these variable effects, one should interpret these results cautiously. However, one might suggest that, in contrast to inhibition deficits that are more profound in people with multiple ACEs, emotion regulation might be severely and negatively affected even with fewer incidents of ACEs. Therefore, in line with Pechtel and Pizzagalli (7), our results indicate that deficits in the affective domain should be particularly attended to when interacting with individuals with ACEs.

Notably, the facial expression Stroop task, an emotion-related task, predicted violent offending among victims of childhood trauma in both studies, whereas the non-emotional color Stroop task did not. In Experiment 1, our findings indicate that the frequency of violent behavior did not significantly correlate with the non-emotional color Stroop effects. However, the degree of violent behavior was strongly correlated with the facial Stroop effects for smile responses but not frown responses. The higher sensitivity of smile responses relative to frowns may be attributed to the fact that smiles imply affiliative intent. That is, smiles are mimicked more readily since they are more acceptable and bear no personal costs, which is in contrast to frowns that are mimicked less as they may be associated with aggression and perceived as socially maladaptive behavior (79). Importantly, these results were replicated in Experiment 2. That is, using a different sample in Experiment 2, we replicated the increase of the facial Stroop for smile responses with violence in the categorical analysis. Therefore, these consistent and replicable findings indicate a critical link between childhood trauma and violence by deficits in emotion regulation.

The differences between inhibition and emotion regulation in predicting violent offending may be explained if one considers the findings of Botdorf et al. (47). Their results showed that risk-taking in a laboratory driving task could be predicted by an emotional but not a cognitive Stroop task. Similarly, adolescents with ACEs may be able to hold inappropriate impulses in check under non-emotional (cool) conditions, but their still-developing emotional regulation systems may be compromised in affective contexts (80). In other words, the tendency of individuals toward violence may relate to their deficits in emotion regulation, tapped into by the facial emotion Stroop effects, but not cognitive inhibition, as measured by the color Stroop task.

### 4.3. Ace and violence are related to each other but not to working memory and intelligence

Confirming previous reports (11, 16), in both samples, we found positive correlations between self-reported ACE and violence. This replicates findings that childhood adversity is related to later violent behavior (21), with offender groups reporting four times more ACEs than non-offenders (16).

Further, our results show that fluid intelligence, as measured by the BEFKI, and WM, as measured by the n-back task, were not significantly associated with a history of childhood adversity. Similarly, participants who reported occasional violent behavior did not show any differences in fluid intelligence and WM compared to those who showed normative violent behavior. Notably, in Experiment 2, we increased the difficulty of the WM test (replacing the one-back with a two-back task) in order to rule out the possibility that the null results found in Experiment 1 were related to the ceiling effect (72). However, as before, neither WM nor fluid intelligence was significantly correlated with ACEs or violent behavior. Although some previous studies [e.g., (37)] found WM decrements among individuals with a history of childhood maltreatment, other studies failed to replicate these results [e.g., (38)]. These findings, together with our results, show that ACEs and violent behavior might not affect intelligence and EFs in general but are related to rather specific deficits in cognitive and especially emotional inhibition.

### 4.4. Modulatory effects of experimental stress

In the present experiment, we had expected that stress would enhance the associations of ACE and violence with measures of cognitive processes. Although we consistently found such associations and could also demonstrate that the stress manipulation worked, stress only enhanced the difference between low and high-violence participants in their congruency effects in the facial Stroop task – specifically for smile responses. This result may relate to the specific sensitivity of individuals with highly violent behavior for situations that enforce affiliative intent by requiring a smile response. Thus, when the experimental situation forces participants with a history of violent behavior, who have just been through a stressful situation, to smile, that is, to express affiliative intent, they may become more vulnerable to interference by incongruent stimuli. Notably, previous studies on stress effects on executive functions have been inconsistent. For instance, even though stress is generally thought to impair executive functions [e.g., (49)], it might benefit some aspects of cognition (50). The highly specific stress effects in the present study align with such inconsistencies. Possibly executive functions are modulated by stress only in some participants and specific situations.

Notably, Casey et al. (81) showed that performance in a task involving inhibition was relatively poor and selectively so in the context of an emotional facial expression (smile) relative to a neutral facial expression. Combined with findings that childhood trauma impairs cognitive functioning, such findings may explain the even further depletion of inhibitory control under an emotional context of the facial expression Stroop task and stress, unlike the non-emotional color Stroop task. Reduced ability to react to stress has been shown to be a vulnerability factor linking childhood trauma and psychological disorders (82).

Under stress, the facial expression Stroop task seemed to tap into emotion regulation ability deficiencies that do not seem to appear with the color word Stroop task. Being a period of heightened hypothalamus-pituitary–adrenal (HPA) axis reactivity (9), adolescence presents changes that are sometimes externalized as heightened stress-induced hormonal responses correlated with high impulsivity and cognitive deficiencies (10). Such responses might be exacerbated by stressful situations, more so among victims of trauma (83, 84). Therefore, the present findings revealing a relationship between stress and deficits in emotion regulation further highlight the vulnerability of adolescents with ACEs and a history of violent behavior to stressful situations.

### 4.5. Limitations and perspectives

The present study was impacted by a number of limitations that could be overcome in future research. Unfortunately, we were not able to carry out endocrinological tests such as cortisol analysis in order to ascertain the effectiveness of the TSST in inducing stress. Although the TSST has been shown in other studies to induce stress [e.g., (85)], it would be reassuring to demonstrate such effects not only indirectly at the performance level as we did here.

Although the findings in the present experiments were illuminating in showing specific relation of the facial Stroop tasks with the amount of violent behavior, due to Covid-19 pandemic-related restrictions, we were confined to populations with relatively limited variance in violence. Future research could study adolescents confirmed and held as violent delinquents and compare them to a normative high-school population. The sensitivity of the face Stroop task to show relationships to violent offending in contrast to its absence for the color Stroop task makes this task or similar emotional conflict paradigms critical tools for future investigations of the mechanisms underlying violence and other kinds of deviant conduct.

Finally, for the facial expression Stroop task, we utilized white faces as stimuli for African participants. Although the expressions of the faces employed are considered to be largely universal, it is conceivable that in the face Stroop task, the same versus other ethnicity is relevant. Hence, future studies could investigate any differences in using faces of different ethnicities and races.

### 4.6. Conclusion

In the present study, we confirmed that emotional and cognitive inhibitions are affected by ACE. Importantly, deficits in emotion regulation, but not in non-emotional inhibition, were predicted by violent behavior in adolescents. Interestingly, the negative impact of emotion regulation deficits on violent behavior was aggravated under stress, specifically in the smile response condition. This result indicates the special vulnerability of individuals prone to violent behavior when their emotion regulation is called for and when they are placed in an affiliative social situation.

## Data availability statement

The original contributions presented in the study are included in the article/Supplementary material, further inquiries can be directed to the corresponding author.

## Ethics statement

The studies involving human participants were reviewed and approved by Kenya’s National Commission for Science, Technology, and Innovation (NACOSTI) and the Kenyatta National Hospital-University of Nairobi – Ethics and Research Committee (KNH/UoN-ERC). Written informed consent to participate in this study was provided by the participants’ legal guardian/next of kin.

## Author contributions

SK: conceptualization, experimental set-up, data collection, data analysis, interpretation, and manuscript writing. WS: conceptualization, interpretation, and manuscript editing. AZ: conceptualization, experimental set-up, data analysis, interpretation, and manuscript editing. All authors contributed to the article and approved the submitted version.

## Funding

The present work was supported by a scholarship by the Deutscher Akademischer Austauschdienst (DAAD) Grant 57460842 to SK. We acknowledge support by the German Research Foundation (DFG) and the Open Access Publication Fund of Humboldt-Universität zu Berlin.

## Conflict of interest

The authors declare that the research was conducted in the absence of any commercial or financial relationships that could be construed as a potential conflict of interest.

## Publisher’s note

All claims expressed in this article are solely those of the authors and do not necessarily represent those of their affiliated organizations, or those of the publisher, the editors and the reviewers. Any product that may be evaluated in this article, or claim that may be made by its manufacturer, is not guaranteed or endorsed by the publisher.

## Footnotes

^1^ www.neurobs.com
