# Supplementary material for: Childhood trauma and violent behavior in adolescents are differentially related to cognitive-emotional deficits
Source: Front Public Health. 2023 Apr 3;11:1001132. doi: 10.3389/fpubh.2023.1001132 (PMC10106606; doi:10.3389/fpubh.2023.1001132)
Supplement: Supplementary file 1 [file Table_1.docx]

**Appendix**

**Table A1**

Experiment 1 ANOVA for Accuracies across Congruent, Incongruent, and Neutral conditions for the SCWT and the facial expression Stroop tasks

| ANOVA | | | | | |
| --- | --- | --- | --- | --- | --- |
| ***Color word Stroop*** | | ***df*** | ***F*** | ***p*** | ***η2*** |
| ***ACE*** | *Congruency* | *2,102* | *14.19*** | *.00* | *.22* |
|  | *Congruency x ACE* | *2,102* | *2.23* | *.11* | *.04* |
| ***VIOLENCE*** | *Congruency* | 2,102 | 12.89** | .00 | .20 |
|  | *Congruency x Violence* | 2,102 | 1.42 | .24 | .03 |
| ***Facial Expression Stroop*** | |  |  |  |  |
| ***ACE*** | *Congruency* | *2,64* | *21.35*** | *.00* | *.40* |
|  | *Congruency x ACE* | *2,64* | *2.86****^†^*** | *.07* | *.08* |
|  | *Response* | *1,32* | *4.33** | *.05* | *.12* |
|  | *Response x ACE* | *1,32* | *3.76****^†^*** | *.06* | *.11* |
|  | *Congruency x Response* | *2,64* | *2.97****^†^*** | *.06* | *.09* |
|  | *Congruency x Response x ACE* | *2,64* | *0.56* | *.57* | *.02* |
|  | *#SMILE: Congruency* | *2,64* | *9.40*** | *.00* | *.23* |
|  | *#SMILE: Congruency x ACE* | *2,64* | *1.27* | *.29* | *.04* |
|  | *#FROWN: Congruency* | *2,64* | *19.1*** | *.00* | *.37* |
|  | *#FROWN: Congruency x ACE* | *2,64* | *2.64* | *.16* | *.08* |
| ***VIOLENCE*** | *Congruency* | *2,64* | *19.75*** | *.00* | *.38* |
|  | *Congruency x Violence* | *2,64* | *0.24* | *.79* | *.01* |
|  | *Response* | *1,32* | *3.96****^†^*** | *.06* | *.11* |
|  | *Response x Violence* | *1,32* | *0.75* | *.39* | *.02* |
|  | *Congruency x Response* | *2,64* | *2.92****^†^*** | *.06* | *.08* |
|  | *Congruency x Response x aggress.* | *2,64* | *O.01* | *.99* | *.00* |

** p < .05; ** p < .01;* ***^†^*** *p < .1*Notes:
# Post-hoc test;

**Table A2**

Experiment 2 ANOVA of accuracies in the SCWT and the facial expression Stroop tasks as a function of Congruency and Stress

| ANOVA | | | | | |
| --- | --- | --- | --- | --- | --- |
| ***Color word Stroop*** | | *df* | *F* | *p* | *η2* |
| ***ACE*** | *Congruency* | *2,120* | *45.53*** | *00* | *.43* |
|  | *Congruency x ACE* | *2,120* | *4.02** | *.01* | *.06* |
|  | *Stress* | *1,60* | *0.16* | *.69* | *.00* |
|  | *Stress x ACE* | *1,60* | *1.09* | *.30* | *.02* |
|  | *Congruency x Stress* | *2,120* | *0.58* | *.56* | *.01* |
|  | *Congruency x stress x ACE* | *2,120* | *1.16* | *.32* | *.02* |
|  | *#Facilitation: Congruency* | *1,60* | *22.68*** | *.00* | *.27* |
|  | *#Facilitation: Congruency x ACE* | *1,60* | *12.36* | *.00* | *.17* |
|  | *#Inhibition: Congruency* | *1,60* | *37.58*** | *.00* | *.39* |
|  | *#Inhibition: Congruency x ACE* | *1,60* | *0.04* | *.84* | *.00* |
| ***Violence*** | *Congruency* | 2,120 | 46.03** | .00 | .43 |
|  | *Congruency x Violence* | 2,120 | 1.86 | .16 | .03 |
|  | *Stress* | 1,60 | 0.24 | .62 | .00 |
|  | *Stress x Violence* | 1,60 | 0.28 | .60 | .00 |
|  | *Congruency x Stress* | 2,120 | 0.78 | .46 | .13 |
|  | *Congruency x Stress x Violence* | 2,120 | 1.05 | .36 | .02 |
| ***Facial Expression Stroop*** | |  |  |  |  |
| ***ACE*** | *Congruency* | *2,120* | *71.40*** | *.00* | *.54* |
|  | *Congruency x ACE* | *2,120* | *6.15*** | *.00* | *.09* |
|  | *Stress* | *1,60* | *0.87* | *.37* | *.01* |
|  | *Stress x ACE* | *1,60* | *3.07****^†^*** | *.09* | *.05* |
|  | *Response* | *1,60* | *12.17*** | *.00* | *.01* |
|  | *Response x ACE* | *1,60* | *10.15*** | *.00* | *.12* |
|  | *Congruency x Stress* | *2,120* | *2.59****^†^*** | *.08* | *.04* |
|  | *Congruency x Stress x ACE* | *2,120* | *0.28* | *.75* | *.01* |
|  | *Congruency x Response* | *2,120* | *1.09* | *.34* | *.02* |
|  | *Congruency x Response x ACE* | *2,120* | *4.60** | *.01* | *.07* |
|  | *Stress x Response* | *1,60* | *1.61* | *.21* | *.03* |
|  | *Stress x Response x ACE* | *1,60* | *0.88* | *.35* | *.01* |
|  | *Congruency x Stress x Response* | *2,120* | *0.53* | *.59* | *.01* |
|  | *Congruency x Stress x Response x ACE* | *2,120* | *0.42* | *.66* | *.01* |
|  | *#SMILES: Congruency* | *2,120* | *37.70*** | *.00* | *.39* |
|  | *#SMILES: Congruency x ACE* | *2,120* | *10.89*** | *.00* | *.15* |
|  | *#FROWNS: Congruency* | *2,120* | *37.74*** | *.00* | *.39* |
|  | *#FROWNS: Congruency x ACE* | *2,120* | *1.26* | *.29* | *.02* |
| ***Violence*** | *Congruency* | *2,120* | *67.62*** | *.00* | *.53* |
|  | *Congruency x Violence* | *2,120* | *0.79* | *.46* | *.01* |
|  | *Response* | *1,60* | *9.00*** | *.00* | *13* |
|  | *Response x Violence* | *1,60* | *1.11* | *.30* | *.02* |
|  | *Stress* | *1,60* | *0.84* | *.36* | *.01* |
|  | *Stress x Violence* | *2,64* | *0.26* | *.61* | *.00* |
|  | *Congruency x Response* | *2,120* | *1.11* | *.36* | *.02* |
|  | *Congruency x Response x Violence* | *2,120* | *0.30* | *.74* | *.01* |
|  | *Congruency x Stress* | *2,120* | *2.83****^†^*** | *.06* | *.05* |
|  | *Congruency x Stress x Violence* | *2,120* | *1.17* | *.32* | *.02* |
|  | *Response x Stress* | *1,60* | *1.23* | *.27* | *.02* |
|  | *Response x Stress x Violence* | *1,60* | *0.92* | *.34* | *.02* |
|  | *Congruency x Response x Stress* | *2,120* | *0.66* | *.52* | *.01* |
|  | *Congruency x Response x Stress x Violence* | *2,120* | *0.38* | *.69* | *.01* |

** p < .05; ** p < .01*Notes:
# Post-hoc test;
